# Echinococcal Disease of the Fallopian Tube as a Rare Cause of Primary Subfertility

**DOI:** 10.7759/cureus.46198

**Published:** 2023-09-29

**Authors:** Shazia Mohammad, Ketav S Joshi, Shaikh Muneeba, Neema Acharya, Shubhada S Jajoo

**Affiliations:** 1 Obstetrics and Gynaecology, Jawaharlal Nehru Medical College, Datta Meghe Institute of Higher Education and Research, Wardha, IND

**Keywords:** echinococcus granulosus, subfertility, fallopian tube, anaphylaxis, hydatid cyst

## Abstract

Echinococcosis is a significant zoonotic infection caused by *Echinococcus granulosus*, which has a worldwide distribution. In India, the annual incidence varies from one to 200 in 100,000 people. The liver and lungs are often affected, making diagnosis difficult when infections occur in uncommon areas. We report a case of a hydatid cyst in the fallopian tube, which presented as subfertility and was confirmed by radiological imaging.

## Introduction

Hydatid disease is an anthropozoonotic infection that can be transmitted from an animal to a human, or vice versa, under naturally occurring conditions. It is caused by the parasite *Echinococcus granulosus* (*E. granulosus*; common name: dog tapeworm) [1]. Hippocrates and other ancient physicians had described hydatid cysts. In 1695, Hartmann described adult *E. granulosus* in the small intestine of dogs, and in 1782, Goeze identified hydatid cysts as the larval stage. The incidence of this disease is high in regions where sheep and cattle are grown, including Australia, Africa, and South America. It is a big health risk in India and is also common in Europe, China, and the Middle East. Tropical areas are less likely to have this disease than temperate areas. While it is rare, a hydatid cyst in the fallopian tube can occur, often in conjunction with involvement in other organs [2]. Patients may experience nonspecific symptoms or be diagnosed incidentally. The most commonly presented symptom is pain, while larger cysts can cause pressure effects or complications such as infection and rupture. We present a rare occurrence of a fallopian tube hydatid cyst presenting primarily with subfertility.

## Case presentation

A 27-year-old nulligravida presented to the OPD with primary subfertility for one and a half years. She also had complaints of bloating and nausea on and off for the past two to three months. Her menstrual history was normal and regular. The general examination was normal. Per abdominal examination, the abdomen was distended with normal overlying skin. On palpation, a soft mass was felt in the right iliac fossa. During the bimanual examination, a solid cystic mass was detected on the right side, separate from the uterus, which obliterated the right and posterior fornices.

Her laboratory investigations indicated hemoglobin was 11 g/mL, total leucocyte count was 14600/cu.mm, differential leucocyte counts were: neutrophils at 65%, lymphocytes at 20%, and eosinophils at 11%; platelets were 5.75 lacs/cu.mm, suggestive of thrombocytosis and eosinophilia. The erythrocyte sedimentation rate (ESR) and C-reactive protein (CRP) were raised. Results for liver and renal function tests were both within the normal range. The HIV, hepatitis B (HBV), and hepatitis C (HCV) serology results were negative. She had a 3.27 ng/mL anti-mullerian hormone (AMH) level and negative tumor markers.

**Figure 1:**
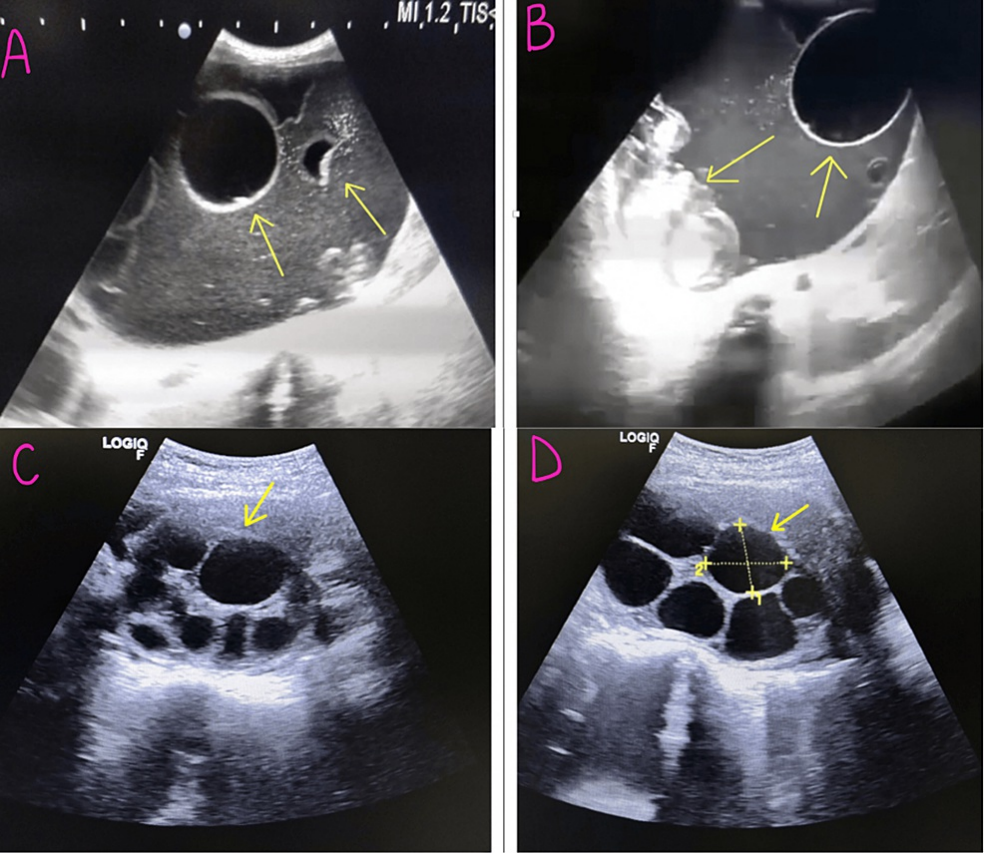
A & B: Ultrasonographic image of liver hydatid cyst showing unilocular cyst; C & D: Hydatid cyst of adnexa showing daughter cysts

Abdomen and pelvis ultrasonography was advised as a part of the infertility workup. Ultrasonography showed evidence of a large anechoic cystic lesion in the right adnexa with septa within the mass of 8.9 x 7 cm, along with a well-defined heterogenous lesion in the right lobe of the liver suggestive of a hydatid cyst of the liver (The WHO-Informal Working Group on Echinococcosis (IWGE) CE1) [3] (Figure 1A, 1B) and a hydatid cyst of the adnexa (WHO-IWGE CE3b) [3] (Figure 1C, 1D).

**Figure 2:**
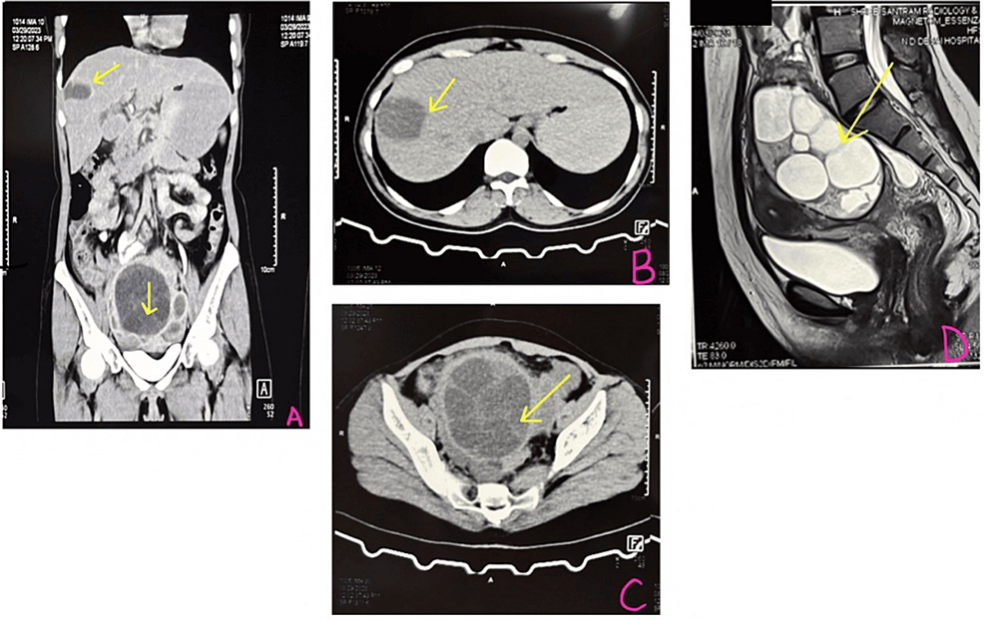
A: Coronal section on CT scan showing liver and pelvic hydatid cysts (yellow arrow); B & C: Axial section on CT scan showing liver and pelvic hydatid cysts, respectively (yellow arrow); D: Sagittal section on a CT scan showing a pelvic hydatid cyst abutting the bowel (yellow arrow) CT: computed tomography

A computed tomography scan (Figures 2A-2D) was done, which showed a large cyst of size 9.2 x 8.6 cm, multivesicular in the pelvic region, and a uniocular cyst in the right lobe of the liver.

The characteristics of the cyst were highly suggestive of hydatid cysts. As there was a high suspicion of the cyst being complicated, albendazole (10 mg/kg) was given for 15 days before surgery.

**Figure 3:**
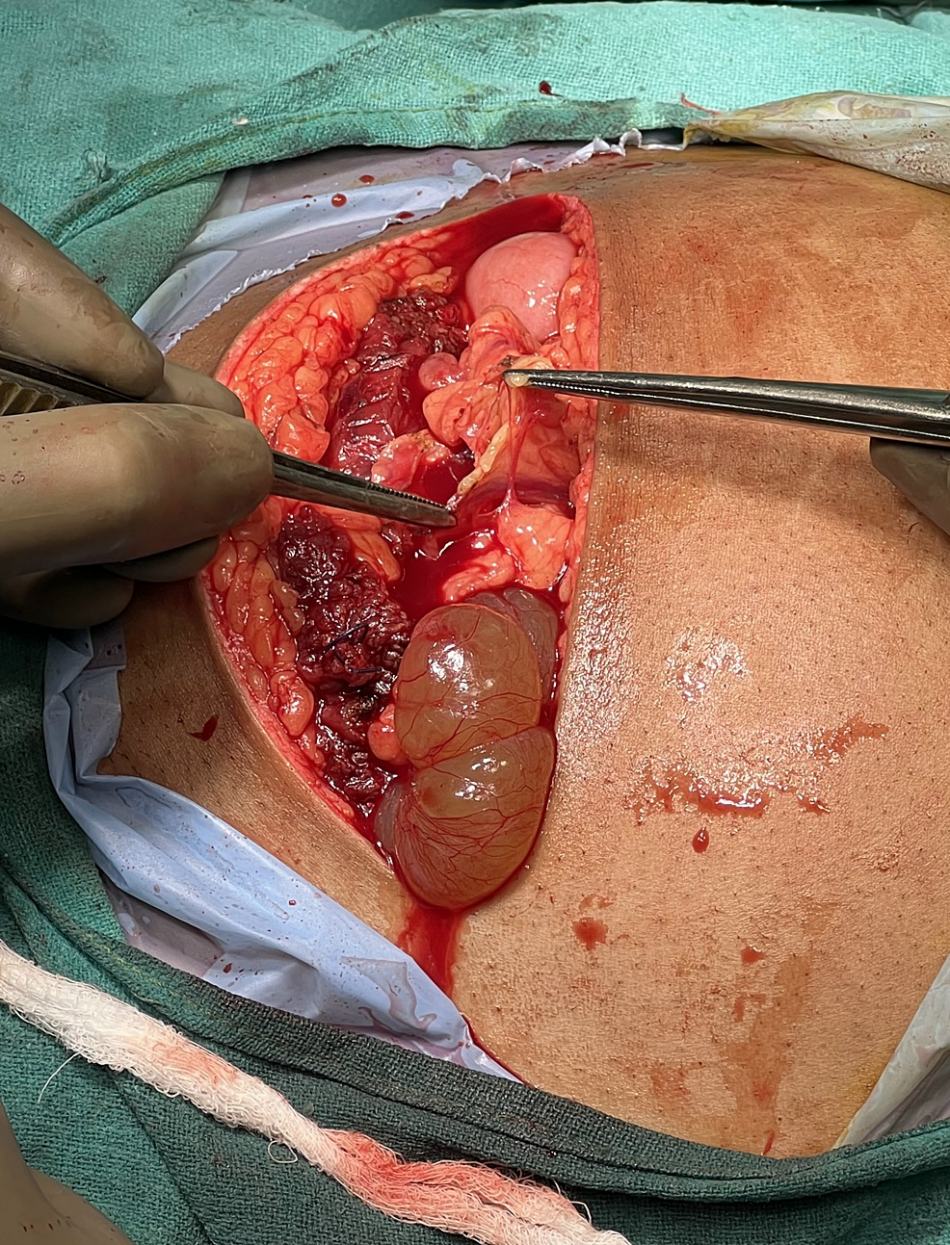
Intraoperative picture of a hydatid cyst

The patient underwent a planned exploratory laparotomy, and it was noted that there were considerable adhesions between the anterior wall of the uterus and omentum and also between the posterior wall of the uterus, omentum, and bowel loops, along with a large hydatid cyst of right tubal origin extending in the pouch of Douglas. Due to considerable adhesions, only the superior aspect of the cyst wall was visible (Figure 3).

**Figure 4:**
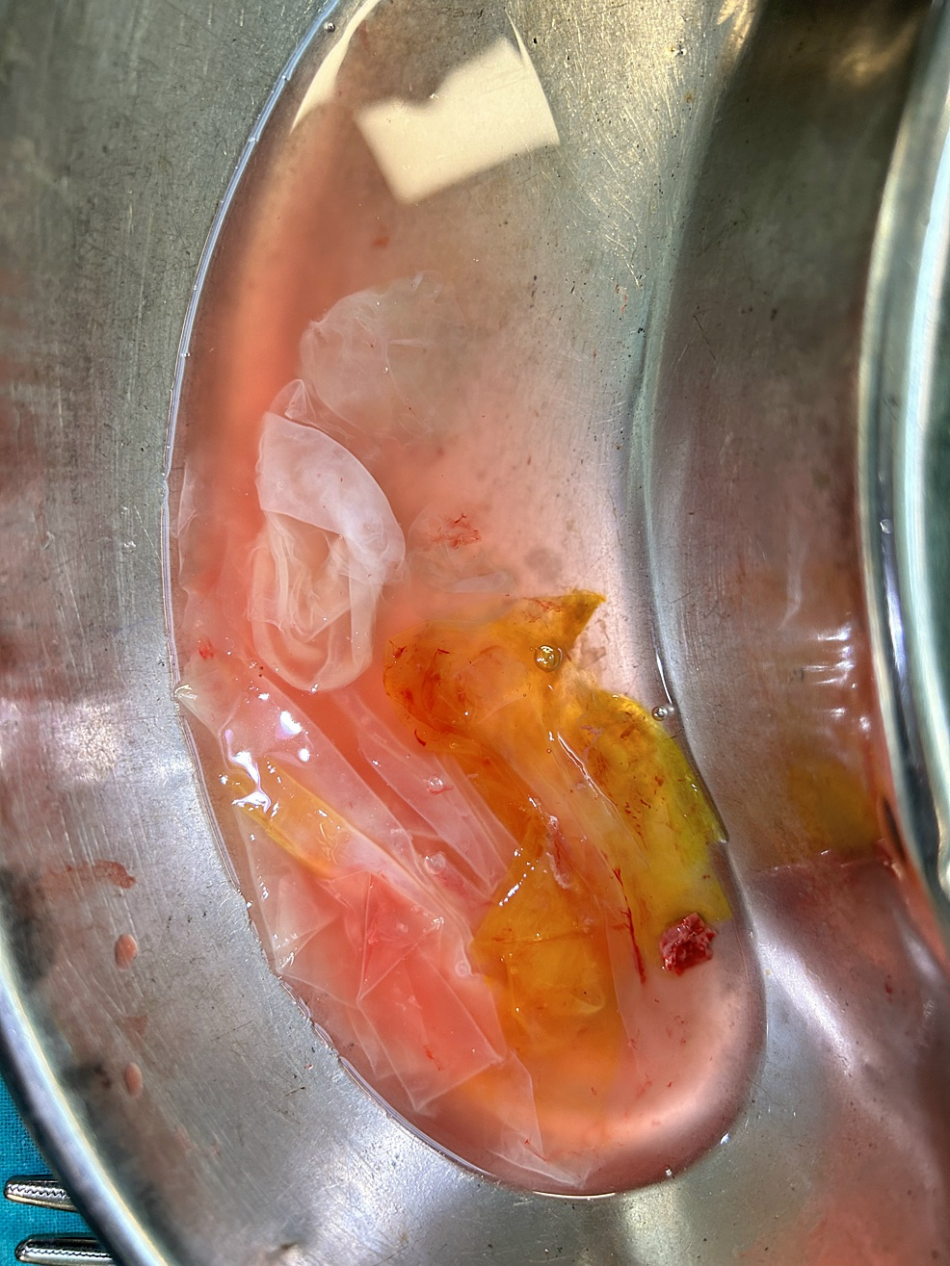
Aspirated daughter cysts

We first removed the omental adhesions. Then bowel loops were freed posteriorly; a segmented bowel loop was still adhered to the cyst wall despite all efforts of adhesiolysis. Meanwhile, we exposed the uterus, fallopian tube, and ovary on the other side. Now the abdominal cavity was packed with surgical mops soaked in hypertonic saline to prevent any spillage of cyst contents. The germinal layer and cyst wall were removed from the superior aspect, and the cyst contents were completely aspirated (Figure 4).

Now, a 20% hypertonic saline solution was injected inside the cystic cavity and kept for 10 minutes to kill the scolices. A right salpingectomy was performed along with segmental bowel resection and anastomosis, which was adhered to the cyst wall. Left-side fallopian tube patency was checked with methylene blue injected by an NGS® Leech Wilkinson intrauterine hysterosalpingography (HSG) cannula, which was found to be patent.

In the same session, liver hydatid cyst removal, hypertonic saline irrigation of the cavity, and omentoplasty were all performed.

Postoperatively, the patient required blood transfusions and ICU admission. However, the patient fully recovered and was released on the tenth postoperative day with a prescription of 400 mg of oral albendazole each day, which was prescribed on discharge for three months. The diagnosis of echinococcal cysts in the liver and fallopian tube was confirmed by the histopathology report.

## Discussion

Life cycle of *Echinococcus granulosus*

**Figure 5:**
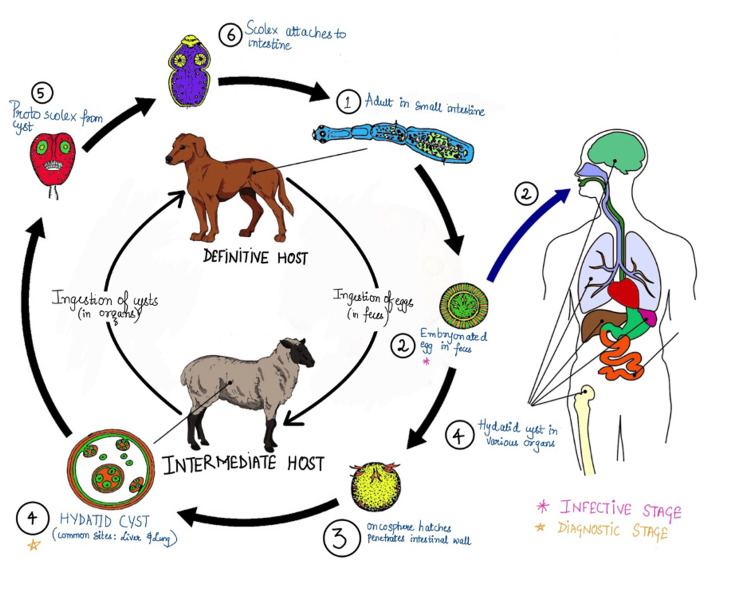
Life cycle of Echinococcus granulosus This image has been created by the author.

The life cycle of *Echinococcus granulosus* involves two types of hosts, with dogs, wolves, jackals, and foxes being the definitive hosts and sheep and other cattle being the intermediate hosts (Figure 5).

The adult form of worm abodes the small intestine of the definitive hosts, which then releases eggs in their feces that are ingested by the intermediate hosts while grazing. Unfortunately, humans can also become accidental intermediate hosts, which brings the life cycle to a halt. The larval stage of the worm is the infectious stage for humans. Human infection occurs when contaminated food items with dog feces containing embryonated eggs are ingested. The eggs ingested by humans are released from the chitinous wall by gastric acid, freeing hexacanth embryo-oncospheres that then penetrate the intestinal wall and enter portal veins, eventually reaching the liver, which is a common site for hydatid cysts. Oncospheres sometimes enter systemic circulation and disseminate in other organs and tissues. These then evolve into infective hydatid cysts. These cysts grow slowly, taking six to 12 months to reach maturity [4].

Pathogenesis

**Figure 6:**
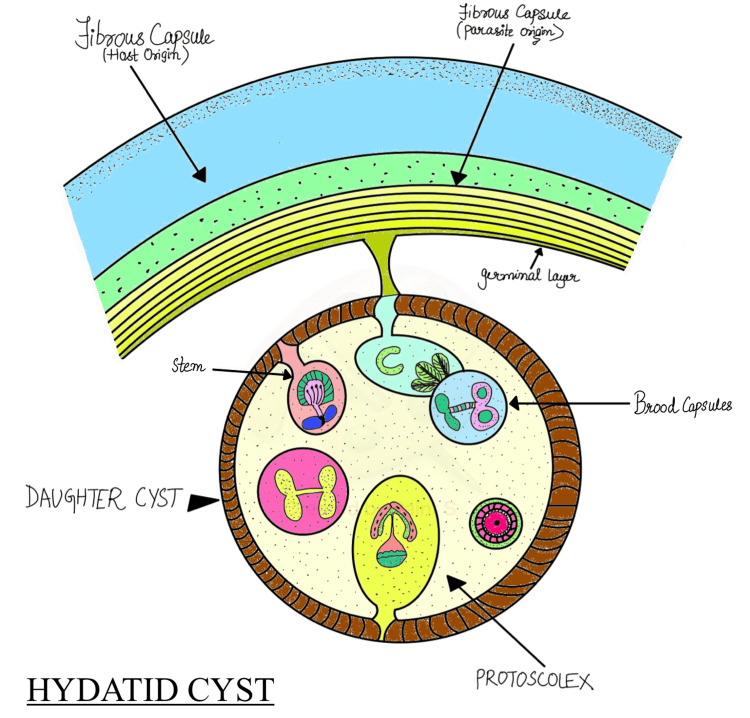
Structure of the hydatid cyst This image has been created by the author.

The embryo gradually develops a cyst at the site of depositing, which fills with fluid to produce the hydatid cyst (Figure 6).

It expands to a diameter of 0.5 to 1 cm after around six months. A fibrous capsule forms around the cyst as a result of the host tissue reacting to the cyst's development. Pericyst, ectocyst, and endocyst are the three indistinguishable layers that make up the cyst wall that the embryo forms. Hydatid fluid, which is clear, colorless, or pale yellow, is present inside the cyst. Hydatid sand, a granular deposit near the cyst's base made up of loose hooklets, protoscolices, and free brood capsules, is present [5].

Clinical manifestations

Clinical manifestations depend on the implicated organ and the severity of the infection. The majority of infections are only discovered at the abattoir. Liver and lung hydatidosis are typically well tolerated without any clinical signs. Pressure from the expanding cyst may result in a range of clinical indications where oncospheres spread to different organs, such as the kidney, pancreas, brain, or marrow of long bones, through the circulation [4,5].

When cysts grow in the cavities of long bones, they can cause various clinical symptoms due to pressure changes. Cysts in the liver can lead to hepatic insufficiency, digestive issues, and ascites, while those in the lungs can cause difficulty breathing. Cysts in the brain can cause paralysis, paraesthesia, blindness, and other cerebral symptoms. If a man is an intermediate host, hydatid in their pulmonary or hepatic sites can result in respiratory distress or abdominal distension, depending on the infected area [4]. The symptoms mainly result from the mechanical pressure of the cyst. Hypersensitivity to echinococcal antigens is another mechanism of pathogenicity. Small amounts of hydatid fluid leak through the capsule and can sensitize the host to the antigen and result in urticaria. However, the massive release of hydatid fluid that occurs when a hydatid cyst ruptures spontaneously or during surgery can result in serious and even fatal anaphylaxis [5].

Diagnostic modalities

When a patient presents with a cyst-like mass and has been exposed to sheep and dogs in an area where hydatidosis is prevalent, a presumptive diagnosis should be made. However, these cysts should be distinguished from other diseases such as benign cysts, cavitary tuberculosis (TB), mycoses, abscesses, and benign or malignant neoplasms.

Radiologic imaging and immunodiagnostic methods can typically be combined to confirm the diagnosis non-invasively. Echinococcal cysts can be found by radiography in the lungs, but calcification is required for visualization elsewhere. Ultrasonography, MRI, and CT scans help determine the extent and status of avascular hydatid cysts as well as for the identification of any dissemination. Abdominal ultrasonography has emerged as the most widely used imaging technique for echinococcosis because of its widespread availability and usefulness for defining the number, site, dimensions, and vitality of cysts [5]. Portable ultrasonography machines have been applied for field surveys with excellent results [6,7].

**Figure 7:**
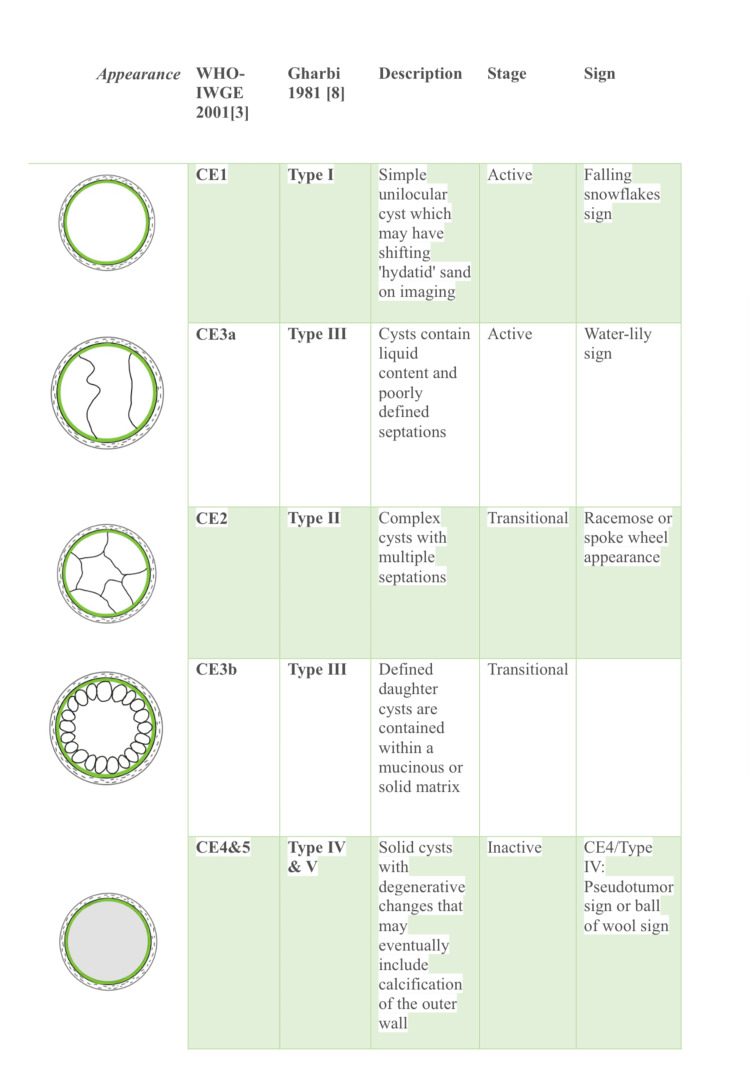
Comparison and interpretation of WHO-IWGE 2001 and Gharbi classification systems for hydatid cyst This image has been created by the author. [3, 8]

There are various systems of staging for hydatid cysts described in the literature. A brief comparison and interpretation of two widely used systems, WHO-IWGE 2001 [3] and Gharbi [8], is described in Figure 7.

While there is no evidence of a detectable immune response in some patients with cystic echinococcosis, antibody tests are helpful to confirm a radiologic diagnosis. Compared to pulmonary cysts, hepatic cysts have a higher likelihood of inducing an immune response. The degree of echinococcal antigen sequestration inside cysts affects the sensitivity of serologic tests regardless of the test's location; for instance, intact cysts may provoke a weak response, whereas previously ruptured or leaking cysts are linked to a strong immune response owing to previous sensitization. For initial serum screening, the enzyme-linked immunosorbent assay (ELISA) has taken the place of the sensitive indirect hemagglutination test. By using immunoblot assays to demonstrate specific echinococcal antigens, reactivity can be specifically confirmed [7]. About 25% of infected people have eosinophilia [9].

In seronegative people, the presence of protoscolices or hydatid membranes in the fluid aspirated from the cyst during percutaneous aspiration can support a presumptive diagnosis. Risks are reduced by using ultrasound guidance for the puncture, using anthelmintics, and anticipating the potential need to manage an allergic reaction [10]. Sometimes protoscolices are seen in sputum or bronchial washings; acid-fast stains make it easier to identify hooklets.

Treatment modalities

Up until the 1980s, the only option available for treating echinococcal cysts was surgery. More recently, however, the use of cyst puncture, aspiration, chemical injection, and re-aspiration has been introduced and is progressively replacing surgery as the preferred treatment. The WHO-IWGE has assessed the advantages and drawbacks of current therapeutic methods [11,12].

Surgery

The treatment with the highest likelihood of completely removing cysts and resulting in an immediate full recovery is still the surgical removal of intact hydatid cysts when possible. Surgery's primary goal is to completely remove the cyst while preventing any negative effects from the cyst's contents spilling. According to the cyst's location and condition, other procedures besides the typical pericystectomy include simple drainage, marsupialization, capitonnage, and resection of the affected organ [12-16]. The more aggressive the course of intervention, the higher the operative risk and, subsequently, the lesser the chances of recurrence, and vice versa. Surgery is the primary treatment whenever liver cysts are more than 10 cm in diameter or secondarily infected [17].

Surgery is contraindicated when patients don’t give consent, are pregnant, have risky pre-existing medical conditions, or have numerous or difficult-to-access cysts. The risks associated with surgery cover both those that are universal (such as anesthesia and infections) and those unique to echinococcosis (like anaphylaxis and secondary recurrence). Operative mortality is 0.5% to 4%; however, it rises with the number of operations and in poor institutions [18].

Chemotherapy

The use of benzimidazole compounds in chemotherapy is now well documented, and many patients can benefit from this course of treatment [11]. A third of individuals receiving benzimidazole medication have been cured (i.e., their cysts have vanished completely and permanently), and even greater percentages (30%-50%) have shown considerable cyst size regression and symptom relief [19-21]. However, 20% to 40% of situations are not favorable. Therapy often works well for small (less than 7 mm in diameter), isolated cysts with a thin adventitial response surrounding them, but not for complicated cysts with several compartments, daughter cysts, or thick, calcified adventitial reactions [9].

Albendazole in doses of 10-15 mg/kg/day and mebendazole in doses of 40-50 mg/kg/day have demonstrated efficacy. Albendazole, however, has produced better outcomes, most likely because of its favorable pharmacokinetics and pharmacodynamics, which make intestinal absorption and cyst penetration easier. Treatment must last at least three months. A small percentage of patients using both medications have experienced adverse responses (neutropenia, liver damage, alopecia, and others) that can be reversed after the medication is stopped. Chemotherapy is contraindicated during pregnancy as well as in cases of chronic liver illness and bone marrow depression. Praziquantel and albendazole together have been used to treat hydatid illness with effectiveness [22,23]. In comparison to albendazole therapy alone, the administration of praziquantel at 50 mg/kg in various regimens (once daily, once weekly, or once every two weeks) resulted in very quick and successful results [22].

Percutaneous aspiration, injection, re-aspiration (PAIR)

A third option for the treatment of hydatid cysts in the liver and some other locations consists of percutaneous puncture using sonographic guidance; aspiration of substantial amounts of the liquid contents; injection of a protoscolicidal agent (e.g., 95% ethanol or hypertonic saline) for at least 15 minutes; and re-aspiration (PAIR, puncture, aspiration, injection, and re-aspiration) [23-25]. When using PAIR, a physician must be ready to handle an allergic response. Concurrent benzimidazole therapy can reduce the risk of secondary echinococcosis brought on by unintentional spilling during this surgery; in fact, combined therapy (PAIR plus albendazole) may produce better outcomes than either chemotherapy or PAIR alone.

The evaluation of the hydatid cyst after therapy is complicated by its obscure character. The best way to determine an objective response to treatment is to repeatedly measure the cyst's size and consistency by computed tomography, magnetic resonance imaging, or ultrasound at three-month intervals. Such monitoring should continue for at least three years because the timing of recurrence is highly varied. The outcome of chemotherapy or PAIR cannot be reliably predicted by a change in the titer of serologic antibody levels on its own.

The following table highlights the unusual presentation of hydatid cysts noted worldwide and the management outline (Table 1).

**Table 1:** Unusual presentation of hydatid cysts and management USG: ultrasonography; CT: computed tomography; MRI: magnetic resonance imaging; FNAC: fine needle aspiration cytology

Unusual site	Presentation	Author	Management
Hydatid cyst of the abdominal wall	Pain with a lump in the right lower abdomen for three months	Moshref and Malaekah, 2021 [26]	USG was performed, followed by CT and MRI. The mass was removed surgically, both preoperatively and postoperatively. Albendazole prescribed
Disseminated hydatid cyst involving the lungs, liver, pelvis, and neck	Abdominal distension for five years, pain in the abdomen for 20 days, fever with shortness of breath and non-productive cough for one week, projectile vomiting for one week	Jadoon et al., 2022 [27]	A CT scan was performed for diagnosis. Exploratory laparotomy was performed with postoperative tazobactum and metronidazole. Unfortunately, the patient died on postoperative day five.
Cervical hydatid cyst	Isolated left posterior neck mass for one year	Rahimi et al., 2023 [28]	Radiological investigations were inconclusive; therefore, an excisional biopsy and total resection of the mass were done.
Thyroid hydatid cyst	An anterior neck mass	El Bousaadani et al., 2016 [29]	Neck USG was done. Thyroidectomy via a classical neck incision was performed with preservation of the parathyroid gland and recurrent laryngeal nerves.
Hydatid disease in the right supraclavicular region	Multiple cystic swelling in the right supra-clavicular region and left supra-clavicular groove for three to four years	Nandy et al., 2012 [30]	X-ray chest and USG-guided FNAC of mass, followed by USG abdomen. Conservative management was instituted with albendazole.

## Conclusions

Pelvic hydatid cysts, specifically tubal cysts, are uncommon. Therefore, an accurate diagnosis requires a high level of suspicion, knowledge of the disease's epidemiology, and the proper use of imaging. This diagnosis should be considered for patients who reside in areas where the disease is widespread or have a history of previous infections. Imaging modalities such as transvaginal sonography and CT scans, particularly ultrasound, are the most effective diagnostic tools. Serologic findings can also provide useful information. Radical cystectomy is the preferred treatment, but it may not be available in some cases. In such situations, partial cystectomy can be helpful. To avoid adverse outcomes, patients should receive anti-helminthic therapy simultaneously.
